# Cytomegalovirus-Induced Coombs-Positive Hemolysis or Drug-Induced Hemolysis in an Immunocompetent Young Adult

**DOI:** 10.7759/cureus.24184

**Published:** 2022-04-16

**Authors:** Mirna S Yacoub, Mahyar Doraji, Sri Yadlapalli

**Affiliations:** 1 Internal Medicine, St. Joseph Mercy Oakland Hospital, Pontiac, USA

**Keywords:** direct antiglobulin test, drug-induced hemolysis, immunocompetent adult, direct coombs test, autoimmune hemolytic anemia (aiha), cytomegalovirus (cmv)

## Abstract

Coombs-positive hemolytic anemia induced by cytomegalovirus (CMV) infection is a rare phenomenon, often not life-threatening in immunocompetent young adults. To date, the pathogenesis of CMV-induced severe hemolysis is still unknown.

Here, we discuss a case of a 22-year-old male without significant past medical history who presented with severe hemolytic anemia that required four units of packed red blood cells. Urinalysis showed microscopic hematuria but urine culture and drug screen reported normal findings. Hemoccult result at the bedside was negative. Abdominal ultrasound and computed tomography (CT) imaging all resulted in normal findings except for splenomegaly measured 18 cm. Hematology was consulted which showed a positive direct Coombs antibody test with 3+ IgG and 3+ complement. Peripheral blood smear showed no evidence of schistocytes or occasional teardrop cells but showed toxic granulations and neutrophils indicating an underlying infection. The patient had a bone marrow biopsy which showed erythroid hyperplasia with a slight increase in sideroblast cells; but revealed no evidence of lymphoma, leukemia, or dysplasia. Infectious workup reported negative findings for HIV and hepatitis panel. However, Epstein-Barr virus (EBV) IgM antibodies to viral capsid antigen (VCA) was reported with a value of greater than 160 U/mL. Polymerase chain reaction (PCR) testing for cytomegalovirus (CMV) DNA detected high titers with 481269 IU/mL. The patient initially received intravenous immunoglobulin (IVIG) therapy for five days, antiviral medication for seven days, and high dose therapeutic corticosteroids resulting in stabilization of his blood hemoglobin (Hb) level.

Infections commonly underlie secondary autoimmune hemolytic anemia (AIHA), or it can also be a result of therapy that further exacerbates the course of AIHA. Possible CMV manifestations inducing severe hemolytic anemia in immunocompetent individuals have received inadequate attention. CMV serology studies are not collected regularly in patients with hemolysis, so the incidence of this disorder might be under-reported. Thus, clinicians should take initiative to consider an underlying infection in the differential diagnosis of hemolytic anemia before opting for invasive procedures such as bone marrow biopsy. Randomized control trials are needed for a conclusive treatment specific to hemolytic anemia induced by CMV.

## Introduction

Autoimmune hemolytic anemia (AIHA) is an acquired, heterogeneous group of diseases that includes warm AIHA, cold agglutinin disease (CAD), mixed AIHA, paroxysmal cold hemoglobinuria, and atypical AIHA. It is mediated by autoantibodies directed against red blood cells (RBCs) causing them to be destroyed [1]. The clinical presentation and treatment of AIHA are influenced by several factors, including the type of AIHA, degree of hemolysis, underlying diseases, presence of concomitant comorbidities, and bone marrow compensatory abilities [1].

Infection with cytomegalovirus (CMV), a common member of herpesviridae is very common among populations. In fact, previous studies have shown that 60-100% of the world population is seropositive for CMV [2]. The morbidity and mortality linked to CMV infection in immunocompromised patients, specifically among those with HIV infection and transplant recipients are well studied. It is responsible for a broad range of clinical manifestations related to direct cytotoxic effect of the virus on specific organs such as the gastrointestinal tract, central nervous system, retina, respiratory tract, and hematopoietic system [3]. Severe CMV-induced hemolytic anemia is a very rare phenomenon described mostly in immunocompromised adults and children [3]. It has been reported in two forms: Coombs positive and Coombs negative [4,5]. The pathogenesis of CMV-induced hemolysis is still unknown.

Primary CMV infection in immunocompetent patients appears as an undifferentiated viral syndrome or mononucleosis-like syndrome in which symptoms tend to be benign and self-limited [3]. This case report presents the underlying etiology of Coombs-positive AIHA from a possible CMV infection rather than drug-induced hemolysis.

## Case presentation

A 22-year-old male with no past medical history, only on emtricitabine/tenofovir prophylaxis (started three months prior to hospital admission), presented to the emergency department (ED) with generalized weakness, dyspnea on exertion, and multiple episodes of syncope at home.

Ten days prior to the patient’s presentation at the ED, he was admitted to a community hospital due to four days of cramping abdominal pain, which was initially located around the umbilical area and then radiated to right lower quadrant. It was mostly exacerbated with walking and standing. It was associated with loose watery diarrhea, fatigue, chills, and fever that measured up to 100.3°F. The patient was suspected to have acute appendicitis; however, his blood work, abdominal ultrasound, and computed tomography (CT) imaging had normal findings. He was discharged with oral cephalosporin and metronidazole. Since the discharge, he continued to have worsening fatigue, lightheadedness on standing, palpitations, loss of appetite, nausea, and two to three episodes of dark yellow emesis.

On admission to the ED, significant objective findings reported fever with a temperature of 103.0°F, tachycardia with a heart rate of 114 beats per minute, and hypoxia with an O_2_ saturation of 90% in which he was placed on 2 L of oxygen via nasal cannula. The patient had obese features with BMI of 40.04 kg/m^2^. He was alert and oriented. Head examination was normocephalic and atraumatic. Eye examination was significant for conjunctival pallor with no sign of sclera icterus. Throat examination showed a moist mucus membrane and neck examination reported no signs of lymphadenopathy. The lungs were clear to auscultation bilaterally. Cardiac auscultation revealed tachycardia with a regular rhythm, S1 and S2 heart sounds heard with no extra heart sounds, murmurs, or rubs. The abdomen was soft and non-tender, with bowel sounds present. There were no rashes, bruises or erythema noted on skin examination. No focal neurological deficits were noted on the neurological examination.

The patient denied any history of gastroesophageal reflux disease (GERD), coronavirus disease 2019 (COVID-19) infection, illicit drug use, or any use of over-the-counter medications. He was vaccinated for COVID-19. He denied any family history of hemolytic anemia, clotting disorders, gastrointestinal bleed, or malignancy. He started on emtricitabine/tenofovir, an antiretroviral medication for HIV prophylaxis for men who have sex with men (MSM), three months prior to hospital admission. The patient reported his last sexual activity was a year ago and maintained the use of condoms. He stated that he had absolutely no opportunity for acquisition of HIV infection recently and had no risk anytime in the recent past. He denied any use of tobacco or alcohol. He reported smoking marijuana once a week. He was a vegetarian who included fish in his diet to supplement vitamin B12.

Initial laboratory workup showed abnormal electrolyte values evident for dehydration, and abnormal complete blood counts (CBC) evident for severe anemia (Table 1). At bedside, hemoccult result was negative. Blood culture showed no microbial growth. Ethanol in blood was negative. The patient also had urinalysis with microscopic reflex which showed 1+ protein, 2+ blood, and 3-4/HPF of RBC evident for microscopic hematuria. Urine culture showed no microbial growth. Urine drug screen reported negative findings.

**Table 1:** Laboratory findings for comprehensive metabolic panel (CMP) and complete blood counts (CBC)

Laboratory test	Value	Reference range
Sodium	129 mEq/L	135-144 mEq/L
Potassium	3.5 mEq/L	3.5-5.3 mEq/L
Chloride	97 mEq/L	98-107 mEq/L
CO_2_	24 mEq/L	21-31 mEq/L
Total bilirubin	3.0 mg/dL	0.3-1.0 mg/dL
Blood urea nitrogen (BUN)	7 mg/dL	7-25 mg/dL
Creatinine	0.69 mg/dL	0.70-1.30 mg/dL
White Blood cells	4.9 k/uL	3.7-11.0 k/uL
Hemoglobin (Hb)	6.3 g/dL	13.0-18.0 g/dL
Hematocrit	17.9%	39.0-50.0%
Red blood cells	1.78 m/uL	4.00-6.00 m/uL
Mean corpuscular volume (MCV)	101 fL	80-99 fL
Platelets	192 k/uL	140-440 k/uL
Lactic acid	3.0 mmol/L	0.5-1 mmol/L
Procalcitonin	0.66 ng/L	<0.1 ng/L

**Figure 1:**
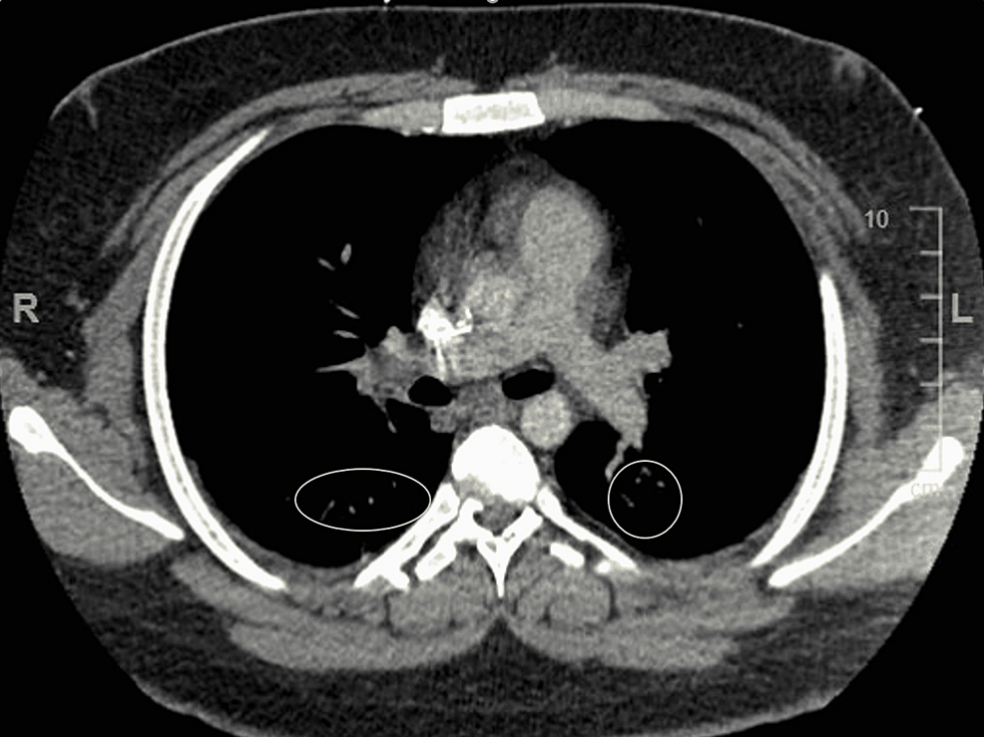
Chest CT angiography showing no acute pulmonary embolism within the central pulmonary arteries Ill-defined ground-glass opacities are shown within the lung bases bilaterally, indicated within the circles.

**Figure 2:**
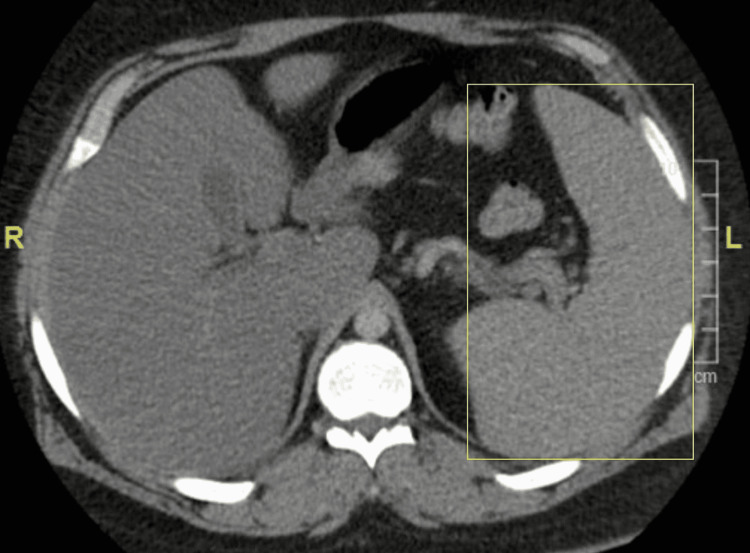
Axial view of abdominal CT angiography showing splenomegaly Enlarged spleen measuring 18 cm shown within the yellow borders.

Given the objective findings of tachycardia and hypoxia, troponin level was unremarkable. Electrocardiogram (EKG) showed sinus tachycardia with no other significant findings which ruled out cardiac effects. Chest CT angiography (CTA) showed no evidence of acute pulmonary embolism but showed ill-defined ground-glass opacities within the base of the lungs bilaterally favoring atelectasis/scarring or less likely representing a multifocal infectious process (Figure 1). CTA also showed hepatic steatosis and splenomegaly measuring up to 18 cm (Figure 2).

The patient was initially started on empiric antibiotics with vancomycin and azithromycin. He also received 4 L of 0.9% IV fluids of isotonic saline due to dehydration. Given the patient’s hemoglobin at 6.3 g/dL which was less than 7.0 g/dL, he received three units of packed red blood cells (PRBC). Blood transfused on admission was screened negative for CMV, HIV, and hepatitis B. He had no history of transfusion or plasma exchange prior to this.

**Figure 3:**
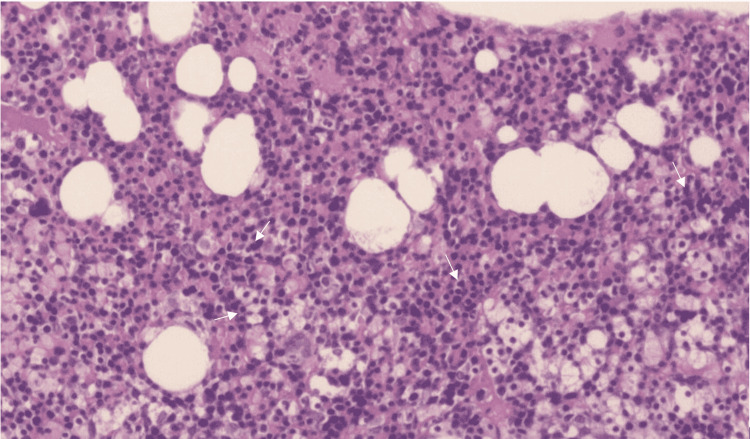
Bone marrow biopsy shows erythroid hyperplasia The image shows groups of erythroid cells (marked with white arrows) with increased cellularity.

Hematology was consulted to further investigate the patient’s severe anemia. Laboratory workup revealed an elevated reticulocyte count with 5.7% (reference value: 0.5-2.0%), decreased haptoglobin with 5 mg/dL (reference value: 41-165 mg/dL), and elevated lactate dehydrogenase with 1215 U/L (reference value: 140-271 U/L). Direct antiglobulin test (DAT)-Coombs antibody showed positive anti-IgG DAT (3+) and anti-complement DAT (3+) with warm antibody indicating warm autoimmune hemolytic anemia. Cold agglutinin was not detected. Folic acid showed normal value but vitamin B12 levels were decreased. A peripheral blood smear showed no evidence of schistocytes or teardrop cells but showed toxic granulations and neutrophils indicating an underlying infection. The patient had a bone marrow biopsy which showed erythroid hyperplasia with a slight increase in sideroblast cells but revealed no evidence of lymphoma, leukemia, or dysplasia (Figure 3).

Within four days of hospital admission, the patient’s baseline hemoglobin (Hb) continued to decline (Table 2). Thus, he received an additional unit of PRBC. Upon hematology consultation, emtricitabine/tenofovir prophylaxis was held off and broad-spectrum antibiotics were discontinued. The patient received intravenous immunoglobulin (IVIG) therapy for five days with continued high-dose corticosteroids leading to stabilization of his Hb level trending up to 8.2 g/dL. Then, he was transitioned to oral corticosteroids of 1 mg/kg body weight.

**Table 2:** Patient's baseline hemoglobin (Hb) within seven days of hospital admission

Day of admission	Hemoglobin values (g/dL)
1	6.3
2	6.9
3	7.2
4	6.6
5	7.2
6	8.1
7	8.2

Given the elevated procalcitonin and lactic acid, infectious disease was consulted (Table 1). HIV Ag/Ab was non-reactive. HIV viral load was not detected. The hepatitis panel was negative. Further workup showed Epstein-Barr virus (EBV) IgM antibodies to viral capsid antigen (VCA) with greater than 160 U/mL. Polymerase chain reaction (PCR) testing for cytomegalovirus (CMV) DNA detected high titers with 481,269 IU/mL. Due to the active infection with CMV, the patient was started on a seven-day course of ganciclovir.

After 13 days of hospitalization, the patient’s Hb was trending up to 9.6 g/dL and was discharged with 120 mg of oral prednisone. He was instructed to follow-up with a hematologist immediately. Two weeks after the last follow-up, the patient’s prednisone regimen was 80 mg daily and was planned on being tapered slowly while monitoring his CBC every two weeks. The patient was not a candidate for rituximab given his high titers of CMV infection at the time. Vitamin B12 supplementation was continued given his vegetarian diet. The patient denied any complaints of fever, chills, syncope, or abdominal pain. He was also following up with infectious disease given his high CMV titers as well as ordering cluster of differentiation (CD)4 counts for further management.

## Discussion

The diagnosis of AIHA can be evaluated with a step-wise approach to identify laboratory and clinical evidence of hemolysis and determine the nature of hemolysis with direct antiglobulin test (DAT) [6]. Investigation with initial elementary findings will first alarm the clinician to the suggestion of hemolysis as the cause of the anemia. Such elementary findings include normo/macrocytic anemia, raised reticulocyte count, raised unconjugated bilirubin, reduced haptoglobin, and blood smear with polychromasia or more specific features, such as spherocytes or agglutination. When hemolysis is confirmed, further investigation is needed to establish whether hemolysis is immune, principally by the direct antiglobulin test (DAT) [6]. One cause of DAT is drug-induced immune hemolysis. A 2015 case report identified 16% of patients (N=73) with drug-induced immune hemolysis caused by ceftriaxone a third-generation cephalosporin [7].

It should not be forgotten that a positive DAT is also a cause of other processes such as viral infections. Extensive studies regarding severe CMV infection in immunocompromised individuals have been reported in biomedical literature. However, manifestations of CMV in immunocompetent individuals have received less attention [2].

In this interesting case report, the patient presented with his symptoms prior to being on cephalosporin antibiotics when it was prescribed upon discharge from the community hospital. His symptomatology continued to worsen with a decline in his baseline Hb until it began to stabilize after administration of prednisone and ganciclovir due to high titers of CMV DNA with 481,269 IU/mL. Also, when a drug is discontinued, hemolytic anemia (HA) resolves soon afterward. Therapeutic with corticosteroids are often not required, and there are limited data to suggest that corticosteroids have any effect when the HA is caused by drug-dependent antibodies [8].

Such finding gives the notion that AIHA was induced by CMV infection. There are only 12 reports of CMV-induced hemolytic anemia which were published between 1980 and 2008. Results showed that the Coombs test was positive in four of them, negative in three, and was not specified for the remaining five patients [3].

Further studies showed that viral load values of 100-300 IU/mL as “low positive” results, whereas a viral load value of 2000-5000 copies/mL correlated with the development of end-organ disease [9]. One case report also showed severe warm autoimmune hemolytic anemia (IgG- and C3d-positive direct antiglobulin test) in an immunocompetent six-month-old infant with acute cytomegalovirus infection [10]. Despite few cases reported in previous years, CMV serology is not regularly collected in patients with hemolysis, therefore the incidence of this disorder is underestimated [10].

Evidence reported in the literature suggests treatment with high dose gamma globulin [11] and a short duration of antiviral medication such as ganciclovir followed by multiple months of glucocorticoid [12]. In this case, IVIG, corticosteroids, and antiviral medication were given to the patient resulting in a significant improvement in his AIHA. However, to achieve an accurate clinical judgment and provide better health care management to patients without missing a diagnosis, clinicians should consider possible CMV infection inducing hemolytic anemia in immunocompetent young adults before obtaining more invasive procedures such as bone marrow biopsy.

## Conclusions

This interesting case of severe life-threatening Coombs-positive hemolytic anemia possibly induced by CMV infection in an immunocompetent young adult is rare and often underestimated. The pathogenesis of this disease remains unknown. Infections usually underlie secondary AIHA, or it can also be a result of therapy that further exacerbates the course of AIHA. Thus, active testing for prompt treatment and prevention of infections are highly recommended. Possible therapeutic treatment with corticosteroids and antiviral medications should be considered to improve hemolysis, although the best treatment strategy remains to be defined.
